# Pharmacogenetics of anticancer drugs in non-Hodgkin lymphomas

**DOI:** 10.1054/bjoc.2001.2130

**Published:** 2001-11

**Authors:** L Loni, M Del Tacca, R Danesi

**Affiliations:** Interdepartmental Centre of Clinical Pharmacology and Experimental Therapeutics, University of Pisa, Italy

**Keywords:** pharmacogenetics, chemosensitivity, drug resistance, non-Hodgkin lymphomas

## Abstract

The variability of tumour responses to chemotherapeutic agents is a topic of major interest in current oncology research. Advances in the knowledge of molecular pathology of cancer make available strategies by which tumour cells can be profiled for their genetic background in order to select anticancer agents that might selectively kill cells in a molecular context that matches the mechanism of action of drugs. The next generation of anticancer treatments might thus be tailored on the basis of the numerous molecular alterations identified in tumour cells of a particular patient. However, to exploit these alterations, it is necessary to understand how they influence the cellular pathways that control the sensitivity or, conversely, resistance to chemotherapeutic agents. The aim of this article is to outline major genetic abnormalities in non-Hodgkin lymphomas that can be used to streamline anticancer drug selection and to underscore the major role of pharmacogenetics, which studies the interactions between genetic background and drug activity, to the prediction of likelihood of response and identification of potential new targets for pharmacological intervention. © 2001 Cancer Research Campaign  http://www.bjcancer.com


*British Journal of Cancer* (2001) **85**(10), 1425–1431
© 2001 Cancer Research Campaign
doi: 10.1054/ bjoc.2001.2130, available online at http://www.idealibrary.com on IDEAL

Review

### Pharmacogenetics of anticancer drugs in non-Hodgkin lymphomas

**L Loni, M Del Tacca and R Danesi**

Interdepartmental Centre of Clinical Pharmacology and Experimental Therapeutics, University of Pisa, Italy

**Summary** The variability of tumour responses to chemotherapeutic agents is a topic of major interest in current oncology research. Advances in the knowledge of molecular pathology of cancer make available strategies by which tumour cells can be profiled for their genetic background in order to select anticancer agents that might selectively kill cells in a molecular context that matches the mechanism of action of drugs. The next generation of anticancer treatments might thus be tailored on the basis of the numerous molecular alterations identified in tumour cells of a particular patient. However, to exploit these alterations, it is necessary to understand how they influence the cellular pathways that control the sensitivity or, conversely, resistance to chemotherapeutic agents. The aim of this article is to outline major genetic abnormalities in non-Hodgkin lymphomas that can be used to streamline anticancer drug selection and to underscore the major role of pharmacogenetics, which studies the interactions between genetic background and drug activity, to the prediction of likelihood of response and identification of potential new targets for pharmacological intervention. © 2001 Cancer Research Campaign http://www.bjcancer.com

**Keywords**: pharmacogenetics; chemosensitivity; drug resistance; non-Hodgkin lymphomas

### NON-HODGKIN LYMPHOMAS: CLINICAL RELEVANCE AND THERAPEUTIC MANAGEMENT

Non-Hodgkin lymphomas (NHLs) represent a clinically heterogeneous group of malignancies arising from B, T or NK-lymphoid cells. Approximately 50 000 new cases of NHLs are diagnosed every year in the United States, with 20 500 estimated deaths, representing 4% of cancer incidence and the seventh cause of death for malignancies in the United States (Groves et al, 2000). For clinical purposes, the division into low, intermediate- and high-grade NHLs is commonly used. Low-grade NHLs are initially treated with radiation therapy or single-agent chemotherapy, including alkylators or nucleoside analogues. However, the treatment of advanced-stage, relapsed low-grade NHLs requires more aggressive combination chemotherapy, including first-generation protocols (cyclophosphamide, vincristine and prednisone (CVP) or cyclophosphamide, doxorubicin, vincristine and prednisone (CHOP)) (Fisher, 2000). The use of monoclonal antibodies, targeted against selected antigens (i.e., CD20), is associated with lower toxicity and higher response rates (Fisher, 2000). Intermediate- and high-grade NHLs are managed with radiotherapy and chemotherapy with CHOP, or second- and third-generation regimens, including MACOP-B (methotrexate, doxorubicin, cyclophosphamide, vincristine, prednisone and bleomycin), m-BACOD (methotrexate, bleomycin, doxorubicin, cyclophosphamide, vincristine and dexamethasone), or ProMACE-CytaBOM (cyclophosphamide, etoposide, cytarabine, vincristine, bleomycin, methotrexate and prednisone), resulting in long-term survival rates of more than 80% (Fisher, 2000).

Despite the advances in the knowledge of molecular pathology of NHLs, histologic criteria still represent the mainstay of classification of NHLs and the choice of systemic chemotherapy is mostly empiric. However, the genetic abnormalities of NHLs will permit in the future the outlook for individual patients to be assessed at diagnosis and treatment may be personalised, if we will be able to establish a relationship between gene expression, disease-specific molecular abnormalities and drug activity. Therefore, the present work reviews the recent advances in the field of molecular genetics of NHLs in order to describe selected molecular pathways involved in the response to anticancer drugs and how pharmacogenetics is going to play a role in rational drug choice in the future.

### MOLECULAR DETERMINANTS OF DRUG SENSITIVITY AND RESISTANCE

### Drug metabolism

The nucleoside analogues fluradabine, gemcitabine, 2-chloro-deoxyadenosine and cytarabine have significant activity in the treatment of NHLs. These prodrugs are phosphorylated by the rate-limiting enzyme deoxycytidine kinase to generate metabolites that inhibit ribonucleotide reductase, resulting in a decrease of endogenous deoxynucleotides with enhanced formation of active drug metabolites and incorporation into the DNA (Dumontet et al, 1999). Drug inactivation may occur by dephosphorylation of active metabolites by cellular 5′-nucleotidase, a major nucleoside dephosphorylating enzyme (Dumontet et al, 1999) or by deamination by cytidine deaminase, whose overexpression confers drug resistance in vitro to CD34 + haematopoietic progenitor cells (Schröder et al, 1996). Prolonged exposure to fluradabine, gemcitabine, 2-chloro-deoxyadenosine and cytarabine resulted in the selection of human leukaemia K562 cells displaying a high degree of cross-resistance to all deoxynucleotide analogues, depending on strong increase in cellular 5′-nucleotidase activity and deoxycytidine kinase down-regulation, resulting in poor accumulation of triphosphate metabolites

*Correspondence to:* R Danesi, 55, Via Roma, 56126 Pisa, Italy.
Phone: +39-050-830148; Fax: +39-050-562020; E-mail: r.danesi@med.unipi.it

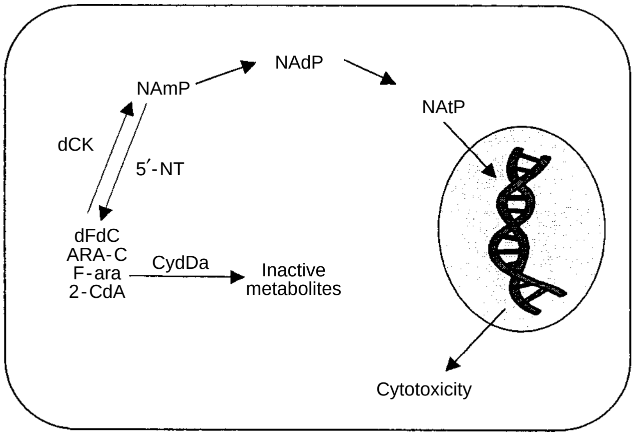

**Figure 1** Relationship between metabolism of the nucleoside analogues gemcitabine (**dFdC**), cytarabine (**ARA-C**), fludarabine (**F-ara**) and 2-chloro-deoxyadenosine (**2-CdA**) in a lymphoma cell and cytotoxicity. Nucleoside analogues (**NA**) are prodrugs that are sequentially activated to the mono-(**NAmP**), di- (**NAdP**) and triphosphate (**NAtP**) metabolites. The anticancer effect of the drugs may be dependent in part on the amount of phosphorylated metabolites produced by **deoxycytidine kinase (dCK)**, the rate-limiting enzyme of nucleoside phosphorylation, whose activity is functionally antagonized by **5′-nucleotidase (5′-NT)**, a cellular dephosphorylating enzyme and by cytidine deaminase (**CydDa**)

(Dumontet et al, 1999). Furthermore, ribonucleotide reductase and cytidine deaminase activities were strongly increased in the gemcitabine-resistant leukaemia K562 cells (Dumontet et al, 1999). CCRF-CEM cell line resistant to hydroxyurea displayed enhanced sensitivity to gemcitabine, due to increased drug uptake and incorporation into DNA as a consequence of enhanced expression of nucleoside transporters (Wong et al, 1999).

In patients with B-CLL resistant to chlorambucil, an increased activity of DNA-dependent protein kinase (DNA-PK), a nuclear serine/threonine kinase that functions in DNA double-strand break repair, was found, suggesting that DNA-PK affects the cellular response to chlorambucil and is involved in the development of nitrogen mustard-resistant disease (Muller et al, 1998). In 20% of anaplastic large cell lymphomas, the fusion of the $NH_2$-terminus of 5-aminoimidazole 4-carboxamide-ribonucleotide formyl-transferase/ IMP cyclohydrolase (ATIC), a bifunctional homodimeric enzyme of de novo purine nucleotide biosynthesis, with the intracellular portion of ALK (anaplastic lymphoma kinase), generates the ATIC-ALK 87 kD chimeric protein, with decreased ATIC activity. This genetic alteration renders lymphomas more sensitive to methotrexate and its analogues, as antifolates inhibit ATIC (Ma et al, 2000). An overview of the metabolic factors affecting drug activity is presented in Figure 1.

### Cellular targets and resistance factors

Follicular lymphomas express the B-cell antigen CD20 on the cell surface (Fisher, 2000); this antigen is the target of the anti-CD20 monoclonal antibody rituximab. Monitoring for the presence of cells harbouring the bcl-2/JH (heavy-chain joining region) gene rearrangement before and after rituximab administration, demonstrated that successful treatment was associated with disappearance of bcl-2/JH rearrangement in cells from bone marrow and peripheral blood (Fisher, 2000). The topoisomerase I inhibitors topotecan and irinotecan have shown activity in NHLs; assessment of expression of topoisomerase I gene can be informative in planning a chemotherapeutic regimen. High levels of topoisomerase I are associated with increase in drug sensitivity; on the contrary, drug resistance of CCRF-CEM cells to topoisomerase I inhibitors results from point mutations of the gene sequence and reduction in the content of nuclear topoisomerase I (Fujimori et al, 1995). Likewise, drug resistance to topoisomerase II inhibitors, including anthracyclines and epipodophyllotoxins, is dependent on point mutations and gene deletions of topoisomerase IIα as well as low expression and alterations in subcellular distribution of the enzyme, as shown in CLL cells (Valkov and Sullivan, 1997).

Proteins encoded by the multi-drug resistance *mdr1* and *mdr* associated (*mrp*) proteins induce chemoresistance in cancer cells to a large number of anticancer agents, including topoisomerase II inhibitors (anthracyclines and epipodophyllotoxins), and vinca alkaloids but not to platinum compounds or alkylating agents. On the contrary, *mdr1-positive* cells are more sensitive or as sensitive as non-*mdr* cells to cytarabine and fludarabine (Michelutti et al, 1997). Patients exposed to high doses of P-glycoprotein transportable drugs show high tumour expression of *mdr1* and *mrp*, as a result of selective pressure of the drugs (Webb et al, 1998). In this setting, chemosensitivity may be restored by P-glycoprotein antagonists, including cyclosporine A and PSC833 (valspodar), although myelosuppression at the bone marrow precursor cell level may be enhanced, as observed with etoposide and cyclosporine A (Lum et al, 2000).

Bryostatin 1, a macrocyclic lactone isolated from the marine bryozoa *Bugula neritina*, is an inhibitor of protein kinase C endowed with antitumour, immune-modulating and differentiating effects on B-cell NHLs. Bryostatin 1 improves the antitumour activity of vincristine in a murine model of diffuse large cell lymphoma, possibly through the down-regulation of *mdr1*/P-glycoprotein and bcl-2 gene expression and up-regulation of p53 (Al-Katib et al, 1998).

It has been demonstrated that the down-regulation of p53 in human leukaemia/lymphoma cell lines resistant to vincristine, may influence *mdr1* overexpression *via* up-regulation of Wilms tumour gene 1 (WT-1) (Hirose and Kuroda, 1998). In patients with NHLs given bifunctional alkylating agents, a significant correlation was observed between complete response to chemotherapy and low expression of glutathione-S-transferase (GST)-α (Ribrag et al, 1996). On the contrary, in patients with CLL, similar levels of GST-α, -μ and -π have been found in normal and neoplastic cells, but patients with low GST-π showed enhanced response to chlorambucil (Ribrag et al, 1996). Increased GST-α and -π isoenzyme expression was reported in CLL resistant to CHOP regimen, compared to CLL sensitive to chlorambucil, while no correlation was found between GST-π expression and P-glycoprotein positivity in CLL (Ribrag et al, 1996). Finally, low levels of the DNA repair enzyme $O^6$-alkylguanine-DNA alkyltransferase in neoplastic T lymphocytes from patients with mycosis fungoides may be predictive of improved clinical response to the new alkylating agent temozolomide, while increased expression and activity of the enzyme represents a marker of resistance (Dolan et al, 1999).

### Oncogenes and regulators of cell cycle – The Bcl-2 family

Chronic lymphocytic leukaemia cells display higher levels of bax, bcl-2 and mdm-2 than normal B cells (Johnston et al, 1997). Following incubation of CLL cells with 2-chloro-deoxyadenosine,

 © 2001 Cancer Research Campaign fludarabine and chlorambucil, an increase in p53 and mdm-2 occurs in cells with wild-type p53, but not in p53-mutated cells highly resistant to chlorambucil and nucleoside analogues, while dexamethasone and vincristine had no effect on mdm-2 (Johnston et al, 1997). Therefore, 2-chloro-deoxyadenosine, fludarabine and chlorambucil induce cytotoxicity in chronic lymphocytic leukaemia cells through a p53-dependent pathway, whereas dexamethasone and vincristine do not (Johnston et al, 1997). This finding may be explained taking into account that p53-dependent induction of apoptosis occurs as a consequence of DNA damage induced by nucleoside analogues and chlorambucil, while vincristine targets microtubules and dexamethasone affects gene transcription (Johnston et al, 1997). An overview of genetic factors involved in drug response is presented in Table 1.

The human pre-B leukaemia cell line 697 overexpressing bcl-2 exhibited strikingly prolonged survival and markedly reduced apoptotic DNA fragmentation when exposed to a large number of drugs, including dexamethasone, methotrexate, cytarabine, etoposide, vincristine, cisplatin and cyclophosphamide (Reed et al, 1994). In addition to this, bcl-2 overexpression induces resistance to nitrogen mustards and camptothecin (Walton et al, 1993), while up-regulation of bcl-xl is associated with resistance to bleomycin, cisplatin, etoposide and vincristine in FL5.12 prolymphoid human cell line (Minn et al, 1995), despite the different mechanisms of action of each agent. In FL5.12 lymphoid cells transduced with bcl-xl and bcl-2, both members provided similar protection against vincristine and vinblastine, whereas bcl-xl was more effective than bcl-2 in preserving cells against etoposide, teniposide, methotrexate, fluorouracil, hydroxyurea and cisplatin cytotoxicity (Simonian et al, 1997). These results indicate that bcl-xl and bcl-2 provide a differential protection against chemotherapy-induced cell death. Finally, the myeloma cell lines 8226, IM-9 and U266 overexpressing bcl-2 are able to survive to doxorubicin and etoposide and resume their proliferation after the drugs are removed (Tu et al, 1996).

Fludarabine down-regulates the expression of bcl-2 in peripheral blood malignant lymphocytes from patients with $CD5^{+}$ B-chronic lymphocytic leukaemia and mantle cell lymphoma; in vitro, drug-induced bcl-2 down-regulation and apoptosis predicted an objective response to fludarabine (Gottardi et al, 1997). Moreover, high bcl-2/bax ratios may be predictive of a drug-resistant phenotype in B-CLL cells and modulation of these proteins by fludarabine is essential for the induction of cell death (Pepper et al, 1999).

In patients with CLL and hairy cell leukaemia (HCL), who were consecutively selected for treatment with fludarabine and 2-chlorodeoxyadenosine, respectively, bcl-2 oncoprotein expression was evaluated in marrow leukaemia cells before treatment. All samples were found to be bcl-2 positive; 83% of CLL and 100% of HCL patients were responsive to purine analogues. These findings show that bcl-2 is overexpressed in almost all cases of CLL and HCL and that bcl-2 overexpression does not predict a poor response to purine analogues in these diseases (Zaja et al, 1998).

Paclitaxel and docetaxel induce bcl-2 phosphorylation, which in turn results in increased levels of free proapoptotic bax protein, and apoptosis in follicular and Burkitt lymphomas, docetaxel being 100-fold more potent than paclitaxel. Both drugs are able to kill tumour cells, even if they express high levels of bcl-2, provided they are in $G_2$–M cycle phase; however, phosphorylation of bcl-2 and apoptosis do not occur in resting CLL cells (Haldar et al, 1997). H9 cells derived from a T-cell lymphoma selected for resistance to high concentrations of azidothymidine (250 μM) displayed overexpression of bcl-2 and were 2 to 10-fold less sensitive to the toxic effects of cisplatin, vincristine, doxorubicin and etoposide, when compared to parental H9 cells, while they retained sensitivity to nucleoside analogues, including cytarabine (Cinatl et al, 1998).

High levels of the antiapoptotic proteins mcl-1 and bag-1 (a bcl-2-binding protein that inhibits apoptosis) have been related to failure to achieve complete remission in patients with chronic lymphocytic leukaemia treated with chlorambucil, fludarabine and 2-chloro-deoxyadenosine (Kitada et al, 1998). In vitro experiments with combinations of cyclophosphamide, fludarabine and mitoxantrone demonstrated that a decrease in mcl-1 and increase in p53 levels correlated with apoptosis in B-chronic lymphocytic leukaemia cells, while the levels of bcl-2 and bax were not modified (Bellosillo et al, 1999). The down-regulation of egr-1 (early growth response gene-1), c-myc, bcl-xl and NF-kB by curcumin, a phenolic extract of the spice turmeric, causes inhibition of cell proliferation of BKS-2 neoplastic B cells (Han et al, 1999). Microenvironmental factors, including activating anti-CD40 antibody, vascular cellular adhesion molecule-1 (VCAM-1), are epigenetic determinants of resistance to etoposide in JLP119 Burkitt lymphoma cells in vitro. Indeed, the activation of surface protein CD40 and interleukin 4 (IL-4) increases bcl-xl protein levels, while cell signalling mediated by VCAM-1 and IL-4 diminished conformational changes in bax protein and prevented the etoposide-induced release of bax from the constitutive bax–bcl-xl complex and occurrence of apoptosis (Taylor et al, 2000). These interactions provide a paradigm for epigenetically induced drug resistance in lymphoma. Overexpression of bax protein in Burkitt lymphoma cells accelerates cell death induced by camptothecin, etoposide, and vinblastine but is without effect on cisplatin and paclitaxel. These results suggest that cell death by selected anticancer drugs correlates with bax expression (Schmitt et al, 1998).

B-chronic lymphocytic leukaemia cells expressing mcl-1, X-linked inhibitor of apoptosis (antiapoptosis protein-XIAP), bag-1 and bcl-2 are highly sensitive to the cyclin-dependent kinase inhibitors, flavopiridol and 7-hydroxystaurosporine. Flavopiridol and 7-hydroxystaurosporine strongly decreased the expression of antiapoptosis genes; on the contrary, expression of the proapoptotic proteins bax and bak was not affected by flavopiridol (Kitada et al, 2000). Flavopiridol-induced decrease in X-linked inhibitor of apoptosis and mcl-1 precedes apoptosis and occurs independently of caspase activation. Finally, in vitro studies on human chronic lymphocytic leukaemia cells incubated with theophylline, a methylxanthine derivative and phosphodiesterase inhibitor, as well as 2-chloro-deoxyadenosine and chlorambucil, demonstrated that bcl-2 was down-regulated and apoptosis was induced by inhibition of intracellular cyclic adenosine monophosphate (cAMP) degradation (Byrd et al, 2000).

Retinoids are able to modulate cell growth and differentiation in a wide variety of human tumour cells. All-trans retinoic acid (ATRA) inhibits proliferation of NHL-B cells; after ATRA exposure, a 50% reduction in the expression of bcl-2 protein was observed, while bax protein levels were up-regulated in ATRA-sensitive NHL-B cells (Sundaresan et al, 1997).

### Cell-cycle-related factors

*p53* gene mutations are associated with decreased sensitivity of human lymphoma cells to DNA-damaging agents (see Table 1). Burkitt lymphoma and lymphoblastoid cell lines, treated with etoposide, nitrogen mustards and cisplatin are arrested in $G_1$ phase if a wild-type p53 is present, while the same agents fail to induce

© 2001 Cancer Research Campaign 

Table 1 Genetic abnormalities in non-Hodgkin lymphomas affecting drug response

| Genetic abnormality | Drug sensitivity | References | Drug resistance | References |
|---|---|---|---|---|
| **p53** mutations | VCR, DEX (B-CLL)<br>Paclitaxel, VCR (BL) | Johnston et al, 1997<br>Fan et al, 1998 | VP-16, $HN_2$, CDDP (BL)<br>CLB, F-ara, CPTs (B-CLL)<br>2-CdA, F-ara, CLB (B-CLL) | Fan et al, 1994<br>Silber et al, 1994<br>Johnston et al, 1997 |
| **p53** deletions | / | / | F-ara, 2-CdA (B-CLL)<br>DOX, CTX (BL) | Döhner et al, 1995<br>Schmitt et al, 1999 |
| **INK4a/ARF** mutations | / | / | CTX (BL) | Schmitt et al, 1999 |
| **ras** overexpression | DEX, VP-16 (Thymic lymphoma) | Kobzdej et al, 2000 | / | / |
| **ras** mutations | / | / | DEX, DOX, melphalan (MM) | Rowley et al, 2000 |
| **bcl-2** overexpression | Paclitaxel, docetaxel (FL, BL)<br>F-ara (B-CLL, MCL)<br>ATRA (FL, B-DLCL)<br>F-ara, 2-CdA (B-CLL, HCL) | Haldar et al, 1997<br>Gottardi et al, 1997<br>Sundaresan et al, 1997<br>Zaja et al, 1998 | Nitrogen mustards, CPT (FL, B-DLCL)<br>DEX, MTX, ARA-C, VP-16, VCR, CDDP, CTX (FL, B-DLCL)<br>DOX, VP-16 (MM)<br>VCR, VLB (FL, B-DLCL)<br>CDDP, VCR, DOX, VP-16 (T-cell lymphoma)<br>DEX (Thymic lymphoma) | Walton et al, 1993<br>Reed et al, 1994<br>Tu et al, 1996<br>Simonian et al, 1997<br>Cinatl et al, 1998<br>Kobzdej et al, 2000 |
| **Mcl-1** overexpression | F-ara, CTX, DHAD (B-CLL) | Bellosillo et al, 1999 | CLB, F-ara, 2-CdA (B-CLL) | Kitada et al, 1998 |
| **Bag-1** overexpression | / | / | CLB, F-ara, 2-CdA (B-CLL) | Kitada et al, 1998 |
| **Bcl-xl** overexpression | / | / | BLM, CDDP, VP-16, VCR (FL, B-DLCL)<br>VCR, VLB, VP-16, VM-26, MTX, hydroxyurea, CDDP, fluorouracil (FL, B-DLCL)<br>VP-16 (BL) | Minn et al, 1995<br>Simonian et al, 1997<br>Taylor et al, 2000 |
| **bax** overexpression | ATRA (FL, B-DLCL)<br>CPT, VP-16, VBL (BL) | Sundaresan et al, 1997<br>Schmitt et al, 1998 | CDDP, paclitaxel (BL) | Schmitt et al, 1998 |
| **c-myc** overexpression | $HN_2$ (BL)<br>Rapamycin (BL) | O'Connor et al, 1991<br>Muthukkumar et al, 1995 | / | / |
| **ATIC-ALK** translocation | MTX (ALCL) | Ma et al, 2000 | / | / |

ALCL: anaplastic large cell lymphoma; B-CLL: B-chronic lymphocytic leukaemia; BL: Burkitt lymphoma; B-DLCL: B-diffuse large cell lymphoma; FL: follicular lymphoma; MCL: mantle cell lymphoma; HCL: hairy cell leukaemia; MM: multiple myeloma; ARA-C: cytarabine; ATRA: all-trans-retinoic acid; BLM: bleomicin; 2-CdA: 2-chloro-deoxyadenosine; CDDP: cisplatin; CLB: chlorambucil; CPT(s): camptothecin(s); CTX: cyclophosphamide; DEX: dexamethasone; DHAD: mitoxantrone; DOX: doxorubicin; F-ara: fludarabine; $HN_2$: mechloretamine; MTX: methotrexate; VCR: vincristine; VLB: vinblastine; VM-26: teniposide; VP16: etoposide.

British Journal of Cancer (2001) 85(10), 1425–1431

© 2001 Cancer Research Campaign

$G_1$ arrest in cells containing a mutant *p53* gene (Fan et al, 1994). The degree of $G_1$ arrest observed with these agents correlated with the rate of p53 and $p21^{Waf1/Cip1}$ protein accumulation; etoposide induced rapid increase of both p53 and $p21^{Waf1/Cip1}$, while nitrogen mustards and cisplatin induced slow accumulation of p53 and no substantial changes in $p21^{Waf1/Cip1}$ levels (Fan et al, 1994). Regardless of the differences in $G_1$ arrest and kinetics of p53 or $p21^{Waf1/Cip1}$ accumulation, all agents induced apoptosis to a greater extent in the wild-type than in the mutant p53 cells (Fan et al, 1994). An inverse relationship between chemosensitivity to nitrogen mustards/cisplatin and etoposide was observed in the mutant p53 lines and this finding correlated with topoisomerase II mRNA levels in the cells (Fan et al, 1994).

The INK4a/ARF locus encodes 2 tumour suppressor genes, designated p16INK4a and p19ARF, which are upstream regulators of the retinoblastoma (Rb) and *p53* genes (Schmitt et al, 1999). INK4a/ARF null lymphomas display low p53 expression despite the presence of a wild-type *p53* gene; they are highly aggressive and resistant to cytotoxic agents, including cyclophosphamide and doxorubicin, although high drug doses might induce p53-independent apoptosis in vitro (Schmitt et al, 1999). Lymphocytes from patients with B-chronic lymphocytic leukaemia with *p53* gene mutations and increased expression of the ERCC1 gene (excision repair cross complementing-1) are able to repair drug-induced DNA-damaged genes and are cross-resistant to chlorambucil, fludarabine, and camptothecins in vitro (Silber et al, 1994). *p53* gene deletion is involved in the lack of response to purine analogues, including fludarabine and 2-chloro-deoxyadenosine, and poor survival in B-chronic lymphocytic leukaemia (Döhner et al, 1995), while paclitaxel and vincristine are capable of inducing apoptosis independent of p53 status in human lymphoblastoid cell lines (Fan et al, 1998).

Protein kinase C inhibitors suppress cell proliferation and are useful in overcoming drug resistance by inhibiting *mdr*-mediated drug efflux. They increase the cytotoxicity of DNA-damaging agents, including platinum complexes, by interfering with cellular repair mechanisms. 7-Hydroxystaurosporine (UCN-01) is a protein kinase C inhibitor that blocks cells in $G_1$ phase by promoting accumulation of dephosphorylated retinoblastoma Rb protein as a consequence of inhibition of cyclin-dependent kinases and increase in the expression of cyclin-dependent kinase inhibitor proteins. Moreover, UCN-01 induces the expression of apoptosis-related surface markers such as annexin and Fas (CD95). UCN-01 prevents the $G_2$ checkpoint arrest in human lymphoma CA46 cells lacking normal p53 function and sensitises cells to the effects of DNA-damaging agents, including cisplatin, etoposide, cyclophosphamide, doxorubicin, as well as prednisone and vincristine (Wilson et al, 2000). Finally, UCN-01 increases fludarabine-induced mitochondrial damage, caspase activation, apoptosis, and suppresses clonogenic survival in bcl-2 overexpressing human leukaemia U937 cells by altering bcl-2 phosphorylation (Harvey et al, 2001).

Cells of thymic lymphoma overexpressing ras/raf genes, developed resistance to dexamethasone as a consequence of the overexpression of bcl-2, while retaining their sensitivity to p53-dependent apoptosis by etoposide (Kobzdej et al, 2000). This finding suggests that glucocorticoid-induced apoptosis involves bcl-2 pathway but not p53 activity. The IL-6-dependent myeloma cell line ANBL6 is sensitive to dexamethasone, doxorubicin, and melphalan. IL-6-driven cell proliferation involves ras-signalling pathways; however, N- or K-ras mutations at codon 12 (N-ras12 and K-ras12) lead to a constitutively active ras protein and IL-6 independent growth and protect ANBL6 cells from apoptosis induced by dexamethasone, doxorubicin and melphalan (Rowley et al, 2000).

Overexpression of c-myc is associated with induction of apoptosis by mechloretamine in the JLP116 Burkitt cell line (O'Connor et al, 1991) and by the immunosuppressive drug rapamycin in the immature B cell lymphoma BKS-2 (Muthukkumar et al, 1995). Finally, fludarabine, is able to target non-dividing cells through a specific decrease in the expression of $STAT_1$ (signal transducer and activator of transcription), a key mediator of cellular responses to cytokines and oncoproteins (Frank, 1999).

### CONCLUSIONS

The developments in molecular medicine suggest that pharmacogenetics will influence the decisions concerning the treatment of selected diseases. With the available information, the likelihood of response to fludarabine of a NHL might be dependent, at least in part, on the following pharmacogenetic profile: (1) low bcl-2/bax ratio (Pepper et al, 1999); (2) high cellular deoxycytidine kinase and low 5′-nucleotidase activities (Dumontet et al, 1999); and (3) wild-type *p53* (Feng et al, 2000), while a favourable profile predicting response to CHOP regimen would include the following: (1) wild-type *p53* (Navaratnam et al, 1998); (2) presence of bcl-2 translocation, in particularly if CHOP is associated with rituximab (Vose et al, 2001); and (3) presence of NPM/ALK chimeric protein (Meguerian-Bedoyan et al, 1997).

Today's specialized techniques of bioinformatics are likely to become routine diagnostic tools in the near future to predict the likelihood of response based on the genome-wide profiling of disease and to select anticancer agents based on genetic abnormalities of the neoplasm to be treated. This prospective implies that the classic approach of standard drug combinations for the treatment of a specific tumour, irrespective of its molecular characteristics, may be abandoned in the future in favour of individually tailored cancer chemotherapy, based on the genetic pattern of disease. Novel technologies provide new avenues of investigation, including real-time PCR and cDNA microarray, and offer powerful tools to elucidate the in vivo molecular events involved in the development and progression of NHLs. Other intriguing issues will be the identification of the optimal drug sequence of treatment in combination regimens and the pharmacological monitoring of drug disposition to prevent possible drug–drug interactions and unexpected toxicities due to polymorphism in drug-metabolizing enzymes. It may be thus hypothesized that bioinformatics may help in the future clinicians in the integration of multiple variables, including disease-specific factors and the mechanism of action of drugs, in the formulation of optimized chemotherapeutic protocols.

### REFERENCES

Al-Katib AM, Smith MR, Kamanda WS, Pettit GR, Hamdan M, Mohamed AN, Chelladurai B and Mohammad RM (1998) Bryostatin 1 down-regulates *mdr1* and potentiates vincristine cytotoxicity in diffuse large cell lymphoma xenografts. *Clin Cancer Res* **4**: 1305–1314

Bellosillo B, Villamor N, Colomer D, Pons G, Montserrat E and Gil J (1999) In vitro evaluation of fludarabine in combination with cyclophosphamide and/or mitoxantrone in B-cell chronic lymphocytic leukemia. *Blood* **94**: 2836–2843

© 2001 Cancer Research Campaign 

Byrd JC, Grever MR, Waselenko JK, Willis CR, Park K, Goodrich A, Lucas MA, Shinn C and Flinn IW (2000) Theophylline, Pentostatin (Nipent), and chlorambucil: a dose-escalation study targeting intrinsic biologic resistance mechanisms in patients with relapsed lymphoproliferative disorders. *Semin Oncol* **2** (**Suppl 5**): 37–40

Cinatl J Jr, Kotchetkov R, Groschel B, Cinatl J, Driever PH, Kabickova H, Kornhuber B, Schwabe D and Doerr HW (1998) Azidothymidine resistance of H9 human T-cell lymphoma cells is associated with decreased sensitivity to antitumor agents and inhibition of apoptosis. *Int J Mol Med* **2**: 685–691

Döhner H, Fischer K, Bentz M, Hansen K, Benner A, Cabot G, Diehl D, Schlenk R, Coy J, Stilgenbauer S, Volkmann M, Galle PR, Poustka A, Hunstein W and Lichter P (1995) p53 gene deletion predicts for poor survival and non-response to therapy with purine analogs in chronic B-cell leukemias. *Blood* **85**: 1580–1589

Dolan ME, McRae BL, Ferries-Rowe E, Belanich M, van Seventer GA, Guitart J, Pezen D, Kuzel TM and Yarosh DB (1999) O6-alkylguanine-DNA alkyltransferase in cutaneous T-cell lymphoma: implications for treatment with alkylating agents. *Clin Cancer Res* **5**: 2059–2064

Dumontet C, Fabianowska-Majewska K, Mantincic D, Callet Bauchu E, Tigaud I, Gandhi V, Lepoivre M, Peters GJ, Rolland MO, Wyczechowska D, Fang X, Gazzo S, Voorn DA, Vanier-Viornery A and Mackey J (1999) Common resistance mechanisms to deoxynucleoside analogues in variants of the human erythroleukaemic line K562. *Br J Haematol* **106**: 78–85

Fan S, el-Deiry WS, Bae I, Freeman J, Jondle D, Bhatia K, Fornace AJ Jr, Magrath I, Kohn KW and O'Connor PM (1994) p53 gene mutations are associated with decreased sensitivity of human lymphoma cells to DNA damaging agents. *Cancer Res* **54**: 5824–5830

Fan S, Cherney B, Reinhold W, Rucker K and O'Connor PM (1998) Disruption of p53 function in immortalized human cells does not affect survival or apoptosis after taxol or vincristine treatment. *Clin Cancer Res* **4**: 1047–1054

Feng L, Achanta G, Pelicano H, Zhang W, Plunkett W and Huang P (2000) Role of p53 in cellular response to anticancer nucleoside analog-induced DNA damage. *Int J Mol Med* **5**: 597–604

Fisher RI (2000) Current therapeutic paradigm for the treatment of non-Hodgkin's lymphoma. *Semin Oncol* **27** (**Suppl 12**): 2–8

Frank DA (1999) STAT signaling in the pathogenesis and treatment of cancer. *Mol Med* **5**: 432–456

Fujimori A, Harker WG, Kohlhagen G, Hoki Y and Pommier Y (1995) Mutation at the catalytic site of topoisomerase I in CEM/C2, a human leukemia cell line resistant to campothecin. *Cancer Res* **55**: 1339–1346

Gottardi D, De Leo AM, Alfarano A, Stacchini A, Circosta P, Gregoretti MG, Bergui L, Aragno M and Caligaris-Cappio F (1997) Fludarabine ability to down-regulate Bcl-2 gene product in CD5+leukaemic B cells: in vitro/in vivo correlations. *Br J Haematol* **99**: 147–157

Groves FD, Linet MS, Travis LB and Devesa SS (2000) Cancer surveillance series: non-Hodgkin's lymphoma incidence by histologic subtype in the United States from 1978 through 1995. *J Natl Cancer Inst* **92**: 1240–1251

Haldar S, Basu A and Croce CM (1997) Bcl2 is the guardian of microtubule integrity. *Cancer Res* **57**: 229–233

Han SS, Chung ST, Robertson DA, Ranjan D and Bondada S (1999) Curcumin causes the growth arrest and apoptosis of B cell lymphoma by downregulation of egr-1, c-myc, Bcl-xl, NF-kappa B, and p53. *Clin Immunol* **93**: 152–161

Harvey S, Decker R, Dai Y, Schaefer G, Tang L, Kramer L, Dent P and Grant S (2001) Interactions between 2-fluoroadenine 9-beta-D-arabinofuranoside and the kinase inhibitor UCN-01 in human leukemia and lymphoma cells. *Clin Cancer Res* **7**: 320–330

Hirose M and Kuroda Y (1998) p53 may mediate the mdr-1 expression via the WT1 gene in human vincristine-resistant leukemia/lymphoma cell lines. *Cancer Lett* **129**: 165–171

Johnston JB, Daeninck P, Verburg L, Lee K, Williams G, Israels LG, Mowat MRA and Begleiter A (1997) P53, MDM-2, BAX, and BCL-2 and drug resistance in chronic lymphocytic leukemia. *Leuk Lymphoma* **26**: 435–449

Kitada S, Andersen J, Akar S, Zapata JM, Takayama S, Krajewski S, Wang HG, Zhang X, Bullrich F, Croce CM, Rai K, Hines J and Reed JC (1998) Expression of apoptosis-regulating proteins in chronic lymphocytic leukemia: correlations with in vitro and in vivo chemoresponses. *Blood* **91**: 3379–3389

Kitada S, Zapata JM, Andreeff M and Reed JC (2000) Protein kinase inhibitors flavopiridol and 7-hydroxy-staurosporine down-regulate antiapopotosis proteins in B-cell chronic lymphocytic leukemia. *Blood* **96**: 393–397

Kobzdej M, Matuszyk J, Ziolo E and Strzadala L (2000) Changes in glucocorticoid-induced apoptosis and in expression of Bcl-2 protein during long-term culture of thymic lymphoma. *Arch Immunol Ther Exp* **48**: 43–46

Lum BL, Kaubisch S, Fisher GA, Brown BW and Sikic BI (2000) Effect of high-dose cyclosporine on etoposide pharmacodynamics in a trial to reverse P-glycoprotein (MDR1 gene) mediated drug resistance. *Cancer Chemother Pharmacol* **45**: 305–311

Ma Z, Cools J, Marynen P, Cui X, Siebert R, Gesk S, Schlegelberger B, Peeters B, De Wolf-Peeters C, Wlodarska I and Morris SW (2000) Inv(2)(p23q35) in anaplastic large-cell lymphoma induces constitutive anaplastic lymphoma kinase (ALK) tyrosine kinase activation by fusion to ATIC, an enzyme involved in purine nucleotide biosynthesis. *Blood* **95**: 2144–2149

Meguerian-Bedoyan Z, Lamant L, Hopfner C, Pulford K, Chittal S and Delsol G (1997) Anaplastic large cell lymphoma of maternal origin involving the placenta: case report and literature survey. *Am J Surg Pathol* **21**: 1236–1241

Michelutti A, Michieli M, Damiani D, Melli C, Ermacora A, Grimaz S, Candoni A, Russo D, Fanin R and Baccarani M (1997) Effect of fludarabine and arabinosylcytosine on multidrug resistant cells. *Haematologica* **82**: 143–147

Minn AJ, Rudin CM, Boise LH and Thompson CB (1995) Expression of Bcl-xl can confer a multidrug resistance phenotype. *Blood* **86**: 1903–1910

Muller C, Christodoulopoulos G, Salles B and Panasci L (1998) DNA-Dependent protein kinase activity correlates with clinical and in vitro sensitivity of chronic lymphocytic leukemia lymphocytes to nitrogen mustards. *Blood* **92**: 2213–2219

Muthukkumar S, Ramesh TM and Bondada S (1995) Rapamycin, a potent immunosuppressive drug, causes programmed cell death in B lymphoma cells. *Transplantation* **60**: 264–270

Navaratnam S, Williams GJ, Rubinger M, Pettigrew NM, Mowat MR, Begleiter A and Johnston JB (1998) Expression of p53 predicts treatment failure in aggressive non-Hodgkin's lymphomas. *Leuk Lymphoma* **29**: 139–144

O'Connor PM, Wassermann K, Saragan M, Magrath I, Bohr VA and Kohn KW (1991) Relationship between DNA cross-links, cell cycle and apoptosis in Burkitt's lymphoma cell lines differing in sensitivity to nitrogen mustard. *Cancer Res* **51**: 6550–6557

Pepper C, Thomas A, Hidalgo De Quintana J, Davies S, Hoy T and Bentley P (1999) Pleiotropic drug resistance in B-cell chronic lymphocytic leukemia-the role of Bcl-2 family dysregulation. *Leuk Res* **23**: 1007–1014

Reed JC, Kitada S, Takayama S and Miyashita T (1994) Regulation of chemoresistance by the bcl-2 oncoprotein in non-Hodgkin's lymphoma and lymphocytic leukemia cell lines. *Ann Oncol* **5**: 61–65

Ribrag V, Massade L, Faussat AM, Dreytus F, Bayle C, Gouyette A and Marie JP (1996) Drug resistance mechanisms in chronic lymphocytic leukemia. *Leukemia* **10**: 1944–1949

Rowley M, Liu P and Van Ness B (2000) Heterogeneity in therapeutic response of genetically altered myeloma cell lines to interleukin 6, dexamethasone, doxorubicin, and melphalan. *Blood* **96**: 3175–3180

Schmitt CA, McCurrach ME, de Stanchina E, Wallace-Brodeur RR and Lowe SW (1999) INK4a/ARF mutations accelerate lymphomagenesis and promote chemoresistance by disabling p53. *Genes Dev* **13**: 2670–2677

Schmitt E, Steyaert A, Cimoli G and Bertrand R (1998) Bax-alpha promotes apoptosis induced by cancer chemotherapy and accelerates the activation of caspase 3-like cysteine proteases in p53 double mutant B lymphoma Namalwa cells. *Cell Death Differ* **5**: 506–516

Schröder JK, Kirch C, Flasshove M, Kalweit H, Seidelmann M, Hilger R, Seeber S and Schütte J (1996) Constitutive overexpression of the cytidine deaminase gene confers resistance to cytosine arabinoside *in vitro*. *Leukemia* **10**: 1919–1924

Silber R, Degar B, Costin D, Newcomb EW, Mani M, Rosenberg CR, Morse L, Drygas JC, Canellakis ZN and Potmesil M (1994) Chemosensitivity of lymphocytes from patients with B-cell chronic lymphocytic leukemia to chlorambucil, fludarabine, and camptothecin analogs. *Blood* **84**: 3440–3446

Simonian PL, Grillot DA and Nunez G (1997) Bcl-2 and Bcl-xl can differentially block chemotherapy-induced cell death. *Blood* **90**: 1208–1216

Sundaresan A, Claypool K, Mehta K, Lopez-Berestein G, Cabanillas F and Ford RJ Jr (1997) Retinoid-mediated inhibition of cell growth with stimulation of apoptosis in aggressive B-cell lymphomas. *Cell Growth Differ* **8**: 1071–1082

Taylor ST, Hickman JA and Dive C (2000) Epigenetic determinants of resistance to etoposide regulation of Bcl-X(L) and Bax by tumor microenvironmental factors. *J Natl Cancer Inst* **92**: 18–23

Tu Y, Xu FH, Liu J, Vescio R, Berenson J, Fady C and Lichtenstein A (1996) Upregulated expression of BCL-2 in multiple myeloma cells induced by exposure to doxorubicin, etoposide, and hydrogen peroxide. *Blood* **88**: 1805–1812

Valkov NI and Sullivan DM (1997) Drug resistance to DNA topoisomerase I and II inhibitors in human leukaemia, lymphoma, and multiple myeloma. *Semin Hematol* **34**: 48–62

Vose JM, Link BK, Grossbard ML, Czuczman M, Grillo-Lopez A, Gilman P, Lowe A, Kunkel LA and Fisher RI (2001) Phase II study of rituximab in combination with CHOP chemotherapy in patients with previously untreated, aggressive non-Hodgkin's lymphoma. *J Clin Oncol* **19**: 389–397

 *© 2001 Cancer Research Campaign*

Walton MI, Whysong D, O'Connor PM, Hockenbery D, Korsmeyer SJ and Kohn KW (1993) Constitutive expression of human Bcl-2 modulates nitrogen mustard and camptothecin induced apoptosis. *Cancer Res* **53**: 1853–1861

Webb M, Brun M, McNiven M, Le Couteur D and Craft P (1998) MDR1 and MPR expression in chronic B-cell lymphoproliferative disorders. *Br J Haematol* **102**: 710–717

Wilson WH, Sorbara L, Figg WD, Mont EK, Sausville E, Warren KE, Balis FM, Bauer K, Raffeld M, Senderowicz AM and Monks A (2000) Modulation of clinical drug resistance in a B cell lymphoma patient by the protein kinase inhibitor 7-hydroxystaurosporine: presentation of a novel therapeutic paradigm. *Clin Cancer Res* **6**: 415–421

Wong SJ, Myette MS, Wereley JP and Chitambar CR (1999) Increased sensitivity of hydroxyurea-resistant leukemic cells to gemcitabine. *Clinical Cancer Research* **5**: 439–443

Zaja F, Di Loreto C, Amoroso V, Salmaso F, Russo D, Silvestri F, Fanin R, Damiani D, Infanti L, Mariuzzi L, Beltrami CA and Baccarani M (1998) BCL-2 immunohistochemical evaluation in B-cell chronic lymphocytic leukemia and hairy cell leukemia before treatment with fludarabine and 2-chloro-deoxy-adenosine. *Leuk Lymphoma* **28**: 567–572

*© 2001 Cancer Research Campaign*
